# Editorial: Novel trends in cultivated or cultured meat research, volume II

**DOI:** 10.3389/fnut.2026.1899562

**Published:** 2026-07-21

**Authors:** Marie-Pierre Ellies Oury, Jean-François Hocquette, Sishir K. Kamalapuram

**Affiliations:** 1 Bordeaux Sciences Agro, Feed and Food, Gradignan, France; 2 Institut National de Recherche pour L'agriculture, L'alimentation et L'environnement (INRAE), Université Clermont Auvergne, VetAgro Sup, UMR1213, Recherches sur les Herbivores, Saint Genès Champanelle, France; 3 Sanjeevani Bioservices Pvt., Ltd., Hyderabad, India

**Keywords:** cell culture media, cellular agriculture, cultivated meat, stem cells, tissue engineering

According to a growing number of scientists, stakeholders and investors, cultivated or cultured meat (CM) continues to attract increasing attention as a potential component of future food systems. However, despite major scientific advances, numerous technical, economic, nutritional, regulatory and societal challenges remain before large-scale commercialization and widespread consumer adoption can occur (1). Current research efforts therefore focus on improving cell sources, culture media, scaffolds, bioprocesses, etc as well as the sensory quality, nutritional properties and sustainability of CM production systems.

Following the success of the first volume published in 2024, this second volume of the Research Topic “*Novel trends in cultivated or cultured meat research*” includes nine articles that illustrate the rapid diversification and growing scientific maturity of the field.

Recchia et al. investigated the generation of bovine induced pluripotent stem cells (iPSCs) from fetal fibroblasts for *in vitro* myogenesis and cultured meat applications. Their work highlighted both the potential and the current limitations of bovine cell reprogramming strategies and muscle differentiation protocols for CM production. The study emphasized the importance of developing robust and stable cellular models suitable for scalable cultivated meat systems.

Egger et al. addressed one of the major bottlenecks limiting the industrial scalability of CM systems, namely the high cost of culture media. The authors explored the use of low-concentrations of press cake protein isolates derived from Styrian oil pumpkin and rapeseed as alternatives to serum albumins. Their results demonstrated that these agricultural co-products could maintain biological activity while supporting cell proliferation, thereby contributing to the development of more sustainable and economically viable culture media formulations.

Haach et al. developed chicken muscle and adipogenic cell cultures for cultivated meat production. Using embryonic stem cells, mesenchymal stem cells, and satellite cells derived from chicken embryos, the authors established differentiated muscle and adipose tissue models designed to reproduce the sensory and nutritional characteristics of conventional poultry meat. This study represents an important contribution toward the development of structured cultivated poultry meat production.

Harrison et al. proposed an innovative strategy for scaffold development based on bacterial cellulose scaffolds derived from brewing waste. Their study illustrated how industrial by-products can be valorized for cultivated meat applications while simultaneously contributing to circular bioeconomy approaches and improving sustainability of scaffold production systems.

Sugama et al. investigated the modulation of the nutritional composition and volatile aroma compounds of cultivated pork fat through culture medium supplementation. Their work demonstrated that modifications to culture conditions can significantly influence both lipid composition and aroma profiles in cultivated fat tissues, thereby opening new opportunities for optimizing the sensory and nutritional quality of future cultivated meat products.

Savyon, Korol et al. investigated the potential of polyphenol salts to reduce the requirement for fibroblast growth factor 2 (FGF2), one of the main cost drivers of cultured meat media. Using bovine embryonic stem cell aggregates, they demonstrated that sodium curcumin could support aggregate growth and mesodermal differentiation under reduced FGF2 conditions, suggesting a promising strategy for lowering culture medium costs while maintaining biological performance.

Savyon, Tawil et al. further addressed the issue of FGF2 cost by developing a low-cost platform for the production of yeast-derived FGF2. Their study demonstrated that low-purity FGF2 produced in *Pichia pastoris* could support bovine embryonic stem cell and mesenchymal stem cell aggregates in suspension culture, exhibiting biological activity comparable to, or even exceeding that of commercial high-purity FGF2.

Hibino examined public perceptions of cell-cultured “meat” in Japan and the United Kingdom, with particular attention to cultural context and the acceptability of referring to these products as “meat.” The study showed that attitudes toward cultured meat are shaped not only by environmental concerns but also by culturally specific perceptions of meat, life, dietary practices and product labeling.

Domagała et al. provided a critical comparative review of cell sources and engineering strategies for cultured meat production. The authors compared fibroblasts, satellite cells, adipocytes, embryonic stem cells, and induced pluripotent stem cells, emphasizing that no single cell type can fully reproduce the complexity of native muscle tissue. They argued that future progress in the field will require integrated approaches combining appropriate cell selection, process optimization, 3D culture systems, co-culture strategies, and careful consideration of regulatory, ethical and economic constraints.

Overall, the articles published in this Research Topic illustrate the rapid diversification and growing scientific maturity of cultivated meat research, particularly with regard to technological advances. Collectively, these contributions demonstrate that the field is evolving beyond proof-of-concept studies toward increasingly applied and technically sophisticated investigations.

As highlighted in the first volume of this Research Topic, consumer perception and societal acceptance remain key determinants for the future development of CM products. Beyond technological feasibility, cultivated meat systems will need to demonstrate nutritional value, sensory quality, affordability and tangible environmental benefits in order to achieve broader acceptance. However, this objective has not yet been fully realized. Indeed, Woelken et al. indicated that further research is needed to substantiate sustainability claims regarding CM (2) due to persistent gaps between available evidence and public claims (3). More recently, Khan et al. confirmed that consumer acceptance and clear regulatory frameworks remain critical prerequisites for the successful commercialization of CM (4).

In conclusion, the articles gathered in this second volume provide valuable insights into the current state of the art of cultivated meat research. They illustrate the highly interdisciplinary nature of research on CM, at the intersection of stem cell biology, tissue engineering, food science, sustainability sciences and consumer research. The last two fields remain comparatively underdeveloped and warrant further attention. We hope that this Research Topic will stimulate further scientific discussions and foster new collaborations aimed at addressing the many remaining scientific, technological, regulatory, and societal challenges surrounding cultivated meat.
